# DAWN: a resource for yielding insights into the diversity among wheat genomes

**DOI:** 10.1186/s12864-018-5228-2

**Published:** 2018-12-17

**Authors:** Nathan S. Watson-Haigh, Radosław Suchecki, Elena Kalashyan, Melissa Garcia, Ute Baumann

**Affiliations:** 1 School of Agriculture, Food and Wine, University of Adelaide, PMB 1, Glen Osmond, 5064 SA Australia; 2 Bioinformatics Hub, School of Biological Sciences, University of Adelaide, Adelaide, SA 5005 Australia; 3 CSIRO Agriculture and Food, Glen Osmond, Locked Bag 2, Adelaide, SA 5064 Australia

**Keywords:** Wheat, Diversity, Variants, Genome, Visualisation

## Abstract

**Background:**

Democratising the growing body of whole genome sequencing data available for *Triticum aestivum* (bread wheat) has been impeded by the lack of a genome reference and the large computational requirements for analysing these data sets.

**Results:**

DAWN (Diversity Among Wheat geNomes) integrates data from the *T. aestivum* Chinese Spring (CS) IWGSC RefSeq v1.0 genome with public WGS and exome data from 17 and 62 accessions respectively, enabling researchers and breeders alike to investigate genotypic differences between wheat accessions at the level of whole chromosomes down to individual genes.

**Conclusions:**

Using DAWN we show that it is possible to visualise small and large chromosomal deletions, identify haplotypes at a glance and spot the consequences of selective breeding. DAWN allows us to detect the break points of alien introgression segments brought into an accession when transferring desired genes. Furthermore, we can find possible explanations for reduced recombination in parts of a chromosome, we can predict regions with linkage drag, and also look at diversity in centromeric regions.

**Electronic supplementary material:**

The online version of this article (10.1186/s12864-018-5228-2) contains supplementary material, which is available to authorized users.

## Background

With advances in technology and the reducing costs of high-throughput sequencing, it has become feasible to sequence the large (≈17 Gbp), polyploid genome of bread wheat (*Triticum aestivum*) and resequencing projects have been undertaken [1] or are currently underway [2, 3]. Ahead of these whole genome sequencing projects, data from thousands of sequenced exomes has become available, predominantly from TILLING populations [4, 5]. While exome capture provides a means to sequence and analyse many more individuals by significantly reducing the sequencing space [6], it is limited to the coding regions for which probes have been designed and is sensitive to GC content. As a result, the coverage of coding regions by exome capture has been shown to be inferior to whole genome sequencing [7]. Furthermore, whole genome sequencing is not only more powerful in detecting exome variants [8] but is capable of capturing structural variation and non-exonic variants [9]. However, this comes at the cost of significantly more resources, not only in terms of sequencing but also the analysis.

Genetic diversity is generally estimated at the population level from SNP data and provides information on the amount of genetic diversity between individuals at the whole genome level, but not on its distribution within the genome. However, genetic diversity goes beyond SNPs and includes indels, introgressions and other structural variation such as copy-number-variation (CNV). These are all known to be important drivers of diversity. Introgressions are often the result of wide crosses with landraces, wild relatives or related species such as rye. These donor species are often more resilient and are good sources of tolerance to various diseases and abiotic stresses such as heat and drought, and have been used extensively in wheat breeding [10, 11]. The ability to access and visualise genetic diversity in detail, from whole chromosomes to individual genes, will enable a better understanding and utilisation of the available diversity in a region of interest, irrespective of scale.

While whole genome sequencing resources are available to the community, their wider utility has been impeded due to several factors: 1) the lack of a high quality, contiguous reference genome assembly and gene annotations; 2) the large computational resource requirements for analysing these data sets effectively and 3) the tools for making that information available in a way that would allow breeders to access information on potentially useful genetic diversity. Addressing these issues is key to closing the gap between research and some of the challenges in plant breeding. The release of the IWGSC RefSeq v1.0 assembly [12], and associated annotations, addresses the first of these issues. Through DAWN (Diversity Among Wheat geNomes) we address the other two issues.

## Implementation

DAWN provides convenient access to almost 1 terabyte of precomputed data. This is derived from a Whole Genome Shotgun (WGS) sequencing project of 17 wheat accessions, including Chinese Spring (CS), 62 exome captures, and RNA-seq data from several tissues and developmental stages from CS. For convenience, it also incorporates annotation data released with the CS IWGSC RefSeq v1.0 assembly, including gene annotations and marker location information. This resource is accessible to the wheat community through a JBrowse [13, 14] genome browser interface hosted at http://crobiad.agwine.adelaide.edu.au/dawn/.

### Reference genome and annotations

DAWN uses the *Triticum aestivum* CS IWGSC RefSeq v1.0 genome assembly as the reference genome [12]. To our knowledge, no currently available genome browsers, including UCSC [15], Ensembl [16], IGV [17], Tablet [18] or JBrowse [13, 14], support the CSI indexing schema. Therefore to enable the visualisation of read alignments from BAM files and variant calls from VCF files we have used a version of the reference genome where each pseudomolecule had been split into two smaller “parts”. To facilitate the conversion of coordinates between full-length pseudomolecules and these “parts” we have developed an online conversion tool (http://crobiad.agwine.adelaide.edu.au/dawn/coord/). IWGSC RefSeq v1.0 is accompanied by several GFF3 files which describe the physical location of gene models (v1.0 and v1.1), transposable elements and markers from various platforms (e.g. Illumina Infinium iSelect 90K and 9K SNP chip markers, DArT markers and several EST and SSR data sets). We pre-processed these files to transform coordinates to match the pseudomolecule “parts” (Additional files 1 and 2) and merged functional annotations into the v1.0 high and low confidence gene models (Additional files 3 and 4). Resulting GFF3 files were validated using GenomeTools v1.5.9 [19].

The CS IWGSC RefSeq v1.0 derived annotation tracks are available to the user under “Annotations/RefSeq v1.0” while the various marker data sets are available as tracks under “Markers”. In addition, the v1.1 gene annotations are available to the user under “Annotations/RefSeq v1.1”. The data from these tracks have been indexed, making genes and markers searchable by name, via the location/search box.

### WGS resequencing data

Whole Genome Shotgun (WGS) resequencing data from 16 bread wheat accessions was obtained from Bioplatforms Australia (BPA) [1, 20] in addition to Chinese Spring WGS Illumina data from the ENA (accession number PRJNA392179) [21]. The data was aligned to the reference genome using minimap2 v2.10 [22]. Alignments with a MAPQ <5 were removed and then further processed to create several data tracks per accessions for visualisation. Approximately 29-50% of raw reads failed to align with a MAPQ ≥5 while 38-56% of raw reads aligned with a MAPQ ≥30 (Additional file 5) These tracks are available to the user under “Resequencing/Whole Genome Shotgun (Illumina)”. The BPA panel includes 11 Australian accessions: Baxter, Chara, Drysdale, Excalibur, Gladius, H-45, Kukri, RAC-875, Westonia, Wyalkatchem and Yitpi, and 5 Northern Hemisphere accessions: AC-Barrie (Canada), Alsen (USA), Pastor (CIMMYT), Volcani-DD-1 (Israel) and Xiaoyan-54 (China). All but Xiaoyan-54 are spring wheat.

For each accession the user can access: 1) “Coverage” tracks for visualising read coverage depth patterns at Kbp to Mbp scales. These show the mean coverage (yellow line) as well as 1 and 2 standard deviations (grey background shading). Regions with read coverage >2*SD from the mean were extracted, then merged if ≤500 bp apart and reported if ≥5 kbp (above the mean) or ≥50 kbp (below the mean) in length (Additional file 6). 2) “Read Alignment” tracks for visualising individual read alignments and alignment mismatches at the 100’s of bp scale. 3) “SNP Coverage” tracks for highlighting mismatches between the read alignments and the CS reference. Vertical lines within the read coverage plot indicate the proportion of reads with mismatches to the CS reference and teardrops shown below the coverage track indicate those positions exceeding 90% alternative bases and at ≥3 reads coverage. This track is particularly useful for identifying haplotype blocks at the Kbp scale. Most tracks transition to a read coverage depth or variant density plots at the Kbp-Mbp scale, when the density of information is too high to be visually meaningful.

### Exome capture data

Public exome capture data for 62 accessions was obtained from the ENA (accession number PRJNA227449) [23] and aligned to the reference genome using the same approach as the WGS data described above. These tracks are available to the user under “Resequencing/Exome (Illumina)”. The data set comprises 6 breeding lines, 29 cultivars, 26 landraces and 1 synthetic hexaploid. As with the WGS resequencing data, “Coverage”, “Read Alignment” and “SNP Coverage” tracks are available for each accession.

### Variant calls and variant density

Variant calling was performed for each accession in the WGS resequencing and exome capture data sets, using a SAMtools v1.8 [24] and BCFtools v1.8 calling pipeline, and is accessible under the “Variant Calls” tracks. These tracks show variant positions as vertical bars, coloured according to the alternative allele. Positions that were reported as indels or triallelic are displayed in black. Variants were classified as either high quality homozygous (PASS), low quality homozygous (LowQualHom), high quality heterozygous (Het) or low quality heterozygous (LowQualHet). A summary of the number of variants for each WGS data set is presented in Table 1. By default, only the homozygous (PASS and LowQualHom) variant calls are displayed. However all classes of variants can be toggled on/off using the “Hide sites not passing filter...” available from the track label of “Variant Calls“ tracks. These tracks are particularly useful for identifying haplotype blocks at the 10’s - 100’s Kbp scale, depending on variant density, and for marker development.

**Table 1:** Number of variants (millions) called per accession from WGS data (% indel calls)

Accession	PASS	Het	LowQualHom	LowQualHet
ACBarrie	15.51 (4.0%)	3.87 (2.1%)	4.85 (6.1%)	3.06 (2.0%)
Alsen	22.90 (3.9%)	2.14 (3.9%)	5.15 (8.2%)	3.31 (3.3%)
Baxter	26.44 (4.6%)	4.99 (2.9%)	3.91 (8.8%)	5.05 (2.4%)
Chara	21.59 (4.6%)	4.31 (2.6%)	3.77 (8.5%)	7.16 (1.4%)
Drysdale	23.29 (4.3%)	2.29 (3.4%)	3.67 (10.1%)	1.80 (3.5%)
Excalibur	22.48 (3.9%)	2.75 (3.1%)	3.69 (9.6%)	5.52 (2.3%)
Gladius	23.90 (4.3%)	2.48 (3.1%)	3.41 (10.8%)	2.53 (2.4%)
H45	20.94 (4.2%)	2.88 (2.9%)	3.76 (9.4%)	4.59 (1.6%)
Kukri	17.21 (3.9%)	4.43 (4.1%)	3.13 (8.9%)	2.69 (3.6%)
Pastor	19.52 (4.2%)	5.44 (2.8%)	4.21 (8.0%)	5.56 (2.2%)
RAC875	29.25 (5.0%)	3.23 (3.9%)	2.75 (11.2%)	2.22 (4.4%)
Volcanii	20.28 (4.0%)	2.81 (3.3%)	5.61 (7.2%)	6.87 (2.5%)
Westonia	20.49 (4.5%)	3.50 (3.7%)	4.11 (8.6%)	4.67 (3.2%)
Wyalkatchem	26.67 (5.0%)	2.69 (3.8%)	3.14 (9.4%)	1.73 (3.7%)
Xiaoyan	15.43 (4.4%)	6.30 (1.7%)	3.62 (8.3%)	11.90 (1.1%)
Yitpi	19.59 (4.6%)	7.84 (1.6%)	3.45 (9.3%)	11.77 (1.1%)
Chinese spring	0.09 (26.4%)	0.43 (6.7%)	0.04 (36.9%)	0.21 (8.8%)

The filter values PASS, Het, LowQualHom and LowQualHet correspond to high quality homozygous, high quality heterozygous, low quality homozygous and low quality heterozygous calls repsectively. Filter values can be used to show/hide that particular type of variant in the “Variant Calls” tracks

A higher-level visualisation of variant calls is provided as “Variant Call Density” tracks, calculated as the number of variant calls per 10 Kbp of non-overlapping windows. Regions with variant density >2*SD from the mean were extracted, then merged if ≤40 kbp apart and reported if ≥500 kbp in length (Additional file 7). When used in concert with the read “Coverage” tracks at the multi-Mbp, it provides a way to differentiate genomic regions which are CS-like (good read coverage and low variant density) from those which are more divergent from CS (good read coverage and high variant density).

### Gene expression data

Chinese Spring RNA-derived data was obtained from URGI [25]. Briefly, it comprised of 5 tissues (grain, leaf, root, spike and stem) at 3 different developmental stages and in 2 replicates. For each tissue and developmental stage we aligned the reads to the reference genome using STAR v2.6.0c [26] and provide access to the resulting data via tracks under “Expression/IWGSC/RNA-seq”, for visualising the read alignments which transition to read coverage depth plots at the 10’s of Kbp scale. A summary of coverage profiles is also available for each tissue (under “Coverage Summary”) to help identify tissue-specific expression patterns using a smaller number of tracks. Unlike other gene expression resources (e.g. Wheat Expression Browser [27]) the information in these tracks cannot be directly compared across different samples (no normalisation performed). However, it still provides an insight into whether genes are potentially expressed and if this may be tissue or stage specific.

### Optimising data for JBrowse tracks

Due to the large size of the wheat genome and the data sets used, the size of index files can become quite large (e.g. ≈40 megabytes for each of the 16 WGS BAM files). Large index files can negatively affect the responsiveness of DAWN, especially when viewing many tracks simultaneously. Before JBrowse can render data for a region of a track to be viewed, it potentially has to download 100’s megabytes of index files. Fortunately, JBrowse offers a feature whereby it loads different index files depending on the currently loaded reference sequence (i.e. chromosome part). To take advantage of this feature, we split BAM, VCF and bigWig files into 43 chromosome parts and index these separately. As a result, the BAM indexes are on average a few hundred kilobytes in size and less than 1.3 megabytes per chromosome part. This reduces the delay until the data is rendered.

### Processing pipeline

The processing pipeline underpinning the DAWN data was implemented using Snakemake v5.1.4 [13]. The 2,149 jobs were executed on a compute cluster containing 2 nodes, each with 72 Intel Xeon E5-2699 v3 CPUs (2.30GHz) and 770 gigabytes RAM. The analysis of the WGS and RNA-Seq data sets took 5.7 CPU years, had a peak memory usage of 300 gigabytes and generated over 11 terabytes of data files (≈800 gigabytes are for JBrowse tracks). The commands used in the processing of this data are available in Additional file 8 with example commands and parameters in Additional file 9.

While users have access to over 800 gigabytes of data files, only a fraction of this is downloaded to a user’s computer. This is made possible by JBrowse’s ability to efficiently retrieve and locally cache information for relatively small subset of data.

## Results and discussion

Below we present examples to demonstrate the utility of DAWN in the investigation of genetic diversity among wheat genomes, opportunities for discovery of new alleles or introgression segments as well as its application for marker development and breeding strategies. The ability to visualise data from several accessions at once, together with gene expression data, marker information and gene annotations provides a powerful resource for investigating genetic diversity among wheat genomes.

### Introgressions

Alien introgressions can be easily spotted with DAWN as decreases in read coverage and probably an associated increase in variant density. For example, a wheat accession that contains an introgression fragment from a distantly related species would show few, if any, sequence reads aligned over the corresponding region of the CS reference genome. Even for the more conserved genic regions we may observe few aligned reads if the sequence divergence is too great for the aligner to accurately place. For introgressed portions of closer relatives, such as durum wheat, the reduction in read alignment affects the non-coding regions more dramatically. That is, we see higher numbers of variant calls in the intergenic regions than in the coding regions. The read alignment coverage and variant density tracks allow easy identification of putative deletions and alien introgressions; their approximate physical size can be inferred from CS. This information can provide valuable insights to QTL-cloning projects since the generation and screening of mapping populations may be unsuccessful if the region harbouring the gene of interest is placed within an introgression fragment and thus unlikely to generate informative recombinants.

#### Stem rust locus *Sr36*

**Fig. 1:**
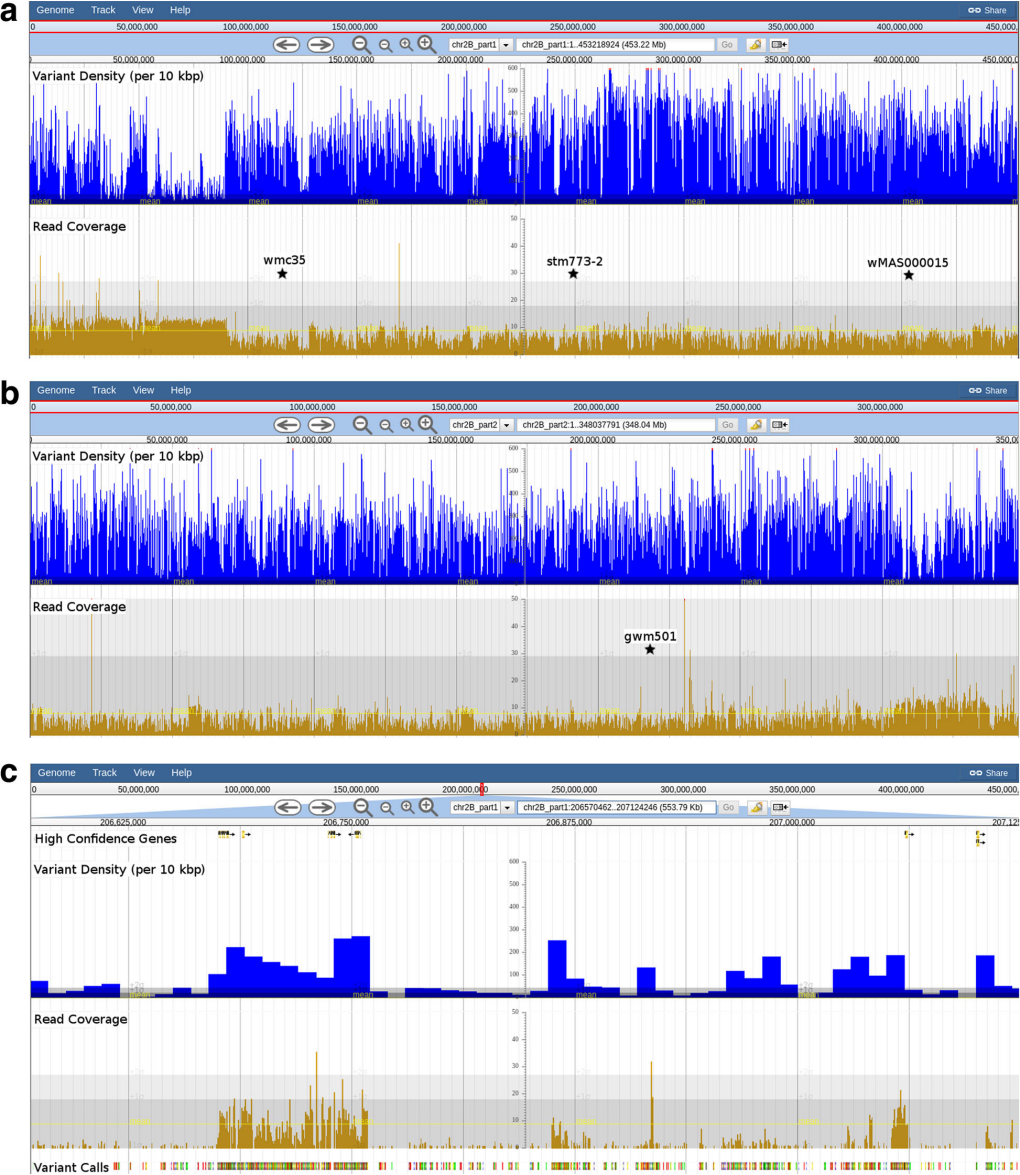
Read coverage depth and variant density for Baxter across chromosome 2B. Read coverage and variant density across the whole of chromosome 2B_part1 (**a**) and chromosome 2B_part2 (**b**) for Baxter. The position of 4 markers discussed in the text are indicated with stars. An example of how read coverage and variant density increase around high confidence (HC) genes is shown together with high quality homozygous SNP/indel calls for chromosome 2B_part1:206570385-207124169 (**c**)

We observed a large region of chromosome 2B in Baxter which showed a consistently reduced read depth coverage and increase variant density compared to the rest of the genome (Fig. 1a and b). The region starts at ≈89.5 Mbp on chr2B_part1, spans the centromeric region, and ends at ≈304.3 Mbp on chr2B_part2. This corresponds to ≈668 Mbp (83%) of the chr2B pseudomolecule and contains 4445 high-confidence gene models. Across this region, we observed increases in read coverage around genes together with increases in variant density (Fig. 1c). This suggested that while much of the intergenic space is very different in Baxter, the gene space is nevertheless similar to the CS reference genome.

The stem rust resistance locus *Sr36*, located on chromosome 2B, is derived from *Triticum timopheevi* and confers resistance against many *Puccinia graminis* sp. *tritici* pathotypes [28]. The microsatellite marker stm773-2 has been found to be tightly linked to *Sr36* and the KASP marker, wMAS000015, is also available [29, 30]. The Australian cultivar, Cook, is derived from the hexaploid wheat CI-12633 which is one of several origins of the *T. timopheeviSr36* introgression [28]. Cook has been used extensively in breeding programs and is a common source of *Sr36* in Australian wheat accessions, including Baxter. A revised genetic map of Sunco (derived from Cook and carrying *Sr36*) x Tasman (not carrying *Sr36*) suggested the *Sr36* translocation extends from marker wmc35 on the short arm, to marker gwm501 on the long arm [31]. By aligning the primer sequences for each of the four markers, to the reference genome, we were able to place wmc35, stm773-2 and wMAS000015 on chromosome 2B_part1 at 113.3 Mbp, 249.7 Mbp and 406.3 Mbp respectively, while gwm501 was placed on chromosome 2B_part2 at 218.9 Mbp (Fig. 1a and b). It has been shown that accessions carrying *Sr36* show no allelic diversity across most of chromosome 2B when compared to accessions which lack it [32], show segregation distortion [33] and linkage repulsion with *Sr39* [34]. This means that combining and introgressing new traits on chromosome 2B in lines possessing *Sr36* derived from CI-12633 will be difficult.

By looking at chromosome 2B in DAWN, we were not only able to find this introgressed region, but were able to delimit the area to a similar interval as previously determined by genetic mapping. However, the *Sr36*-derived gene(s) responsible for stem rust resistance remain elusive, especially given the size of this introgression.

#### Root lesion nematode resistance tightly linked to yellow flour colour

Root lesion nematode (*Pratylenchus neglectus*) infections can cause significant yield losses and thus are a major problem for Australian wheat growers. Moderate resistance has been described for two accessions in the BPA panel, Excalibur and Wyalkatchem, whereas other Australian accessions, including Kukri, Chara, Gladius and Yitpi, are susceptible. The resistance has been attributed to the *Rlnn1* locus located in the terminal region of the long arm of chromosome 7A [35]. Jayatilake et al. [36] showed *Rlnn1* to be tightly linked to the *t* allele of *Psy-A1*, an allele highly associated with yellow pigmentation of the wheat grain. While yellow flour colour is a desirable trait for durum wheats, it is undesirable in bread wheat. Efforts towards separating the loci by recombination have so far been unsuccessful [37].

**Fig. 2:**
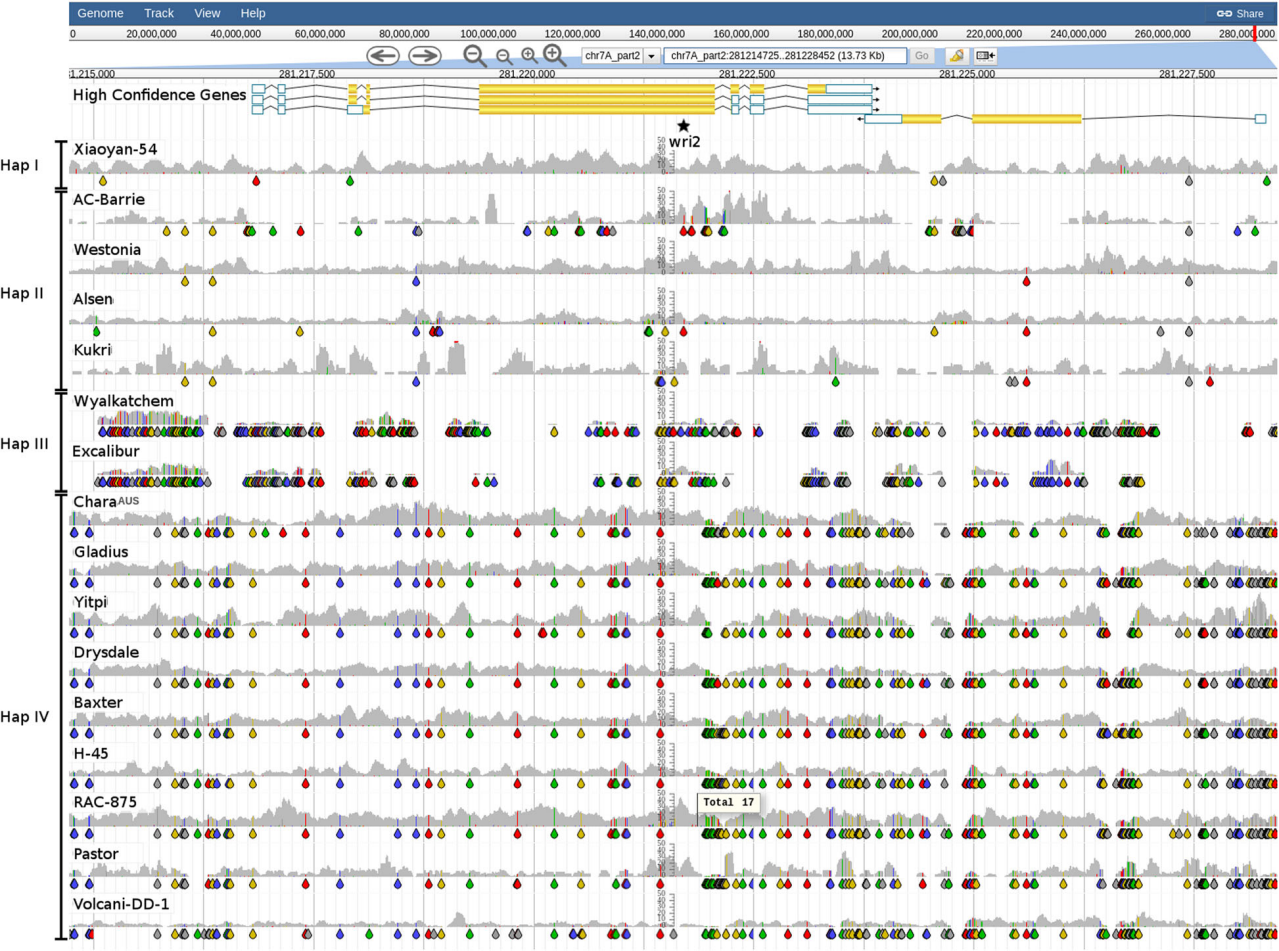
Genomic diversity around the *Rlnn1* marker *wri2*. SNP Coverage plots for the 16 BPA accessions have been sorted into 4 haplotypes based on the SNP/indel patterns in view (chr7A_part2:281214721-281228448). The position of the marker, *wri2*, is indicated by the star. Coloured drops hanging off the read coverage profile indicate the presence of putative SNPs/indels to the reference, A-green, T-red, C-blue, G-yellow, indel-grey

**Fig. 3:**
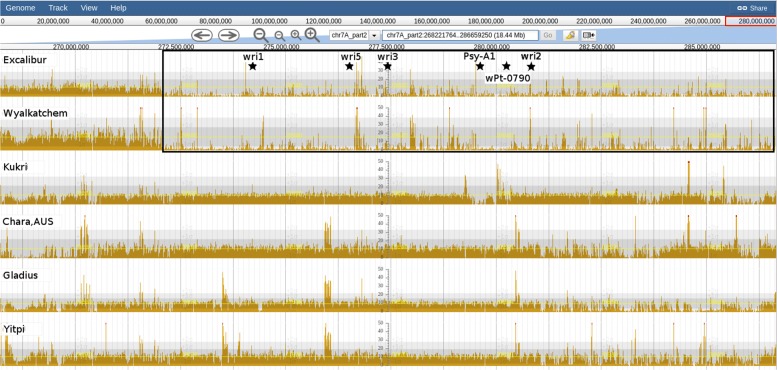
Read alignment coverage for the distal end of chr7A_part2 with positions of markers linked to *Rlnn1*. The positions of several markers, known to be linked to *Rlnn1*, and the location of the *Psy-A1* gene are shown (chr7A_part2:268221621-286659250). The black box highlighting a region of low coverage observed in Excalibur and Wyalkatchem, two accessions known to be moderately resistant to *P. neglectus*, have yellow flour colour and carry *Rlnn1*. For comparison, four other accessions know to be susceptible are included

To investigate what DAWN can reveal about this region, we retrieved sequences for markers (*wri1*, *wri2*, *wri3*, *wri5* and *wPt-0790*) used by Jayatilake et al. [36] and placed these onto the genome by BLASTn. In doing so, we identified TraesCS7A01G557300 as *Psy-A1*. The SNP/indel patterns and read alignment coverage clearly identified at least 4 different haplotypes, with Excalibur and Wyalkatchem being the most different from CS, as evident from few reads being aligned across this region and with higher variant density (Fig. 2). This lack of read alignment coverage extends from position ≈272.1 Mbp on chr7A_part2 to the telomere, i.e. a ≈14.6 Mbp long segment containing 233 high-confidence gene models (Fig. 3). Thus it appears likely that the *Rlnn1* carrying segment has been introgressed from a wild relative of wheat as a terminal substitution. Tight linkage and suppressed recombination observed between *Lr20*/*Sr15* (leaf rust resistance), *Pm1* (powdery mildew resistance) and *Rlnn1* are carried on this introgression and now form part of Excalibur’s and Wyalkatchem’s genomes [38]. Sequence differences between bread wheat and the alien introgression segment likely explains the observed suppressed recombination and the failed attempts to separate *Rlnn1* from the unfavourable *Psy-A1* allele over the last decade.

### Deletions

#### VRN-B1 deletion

Spring and winter growth habit in hexaploid wheat is determined primarily by allelic variation in the *VRN-1* homeologues *VRN-A1* on chromosome 5A, *VRN-B1* on chromosome 5B and *VRN-D1* on chromosome 5D [39, 40]. Briefly, hexaploid spring wheat have a deletion in the first intron of *VRN-B1* and/or *VRN-D1*. Spring types lacking these deletions are expected to have a *VRN-A1* promoter which differs from the recessive *vrn-A1* allele [39].

**Fig. 4:**
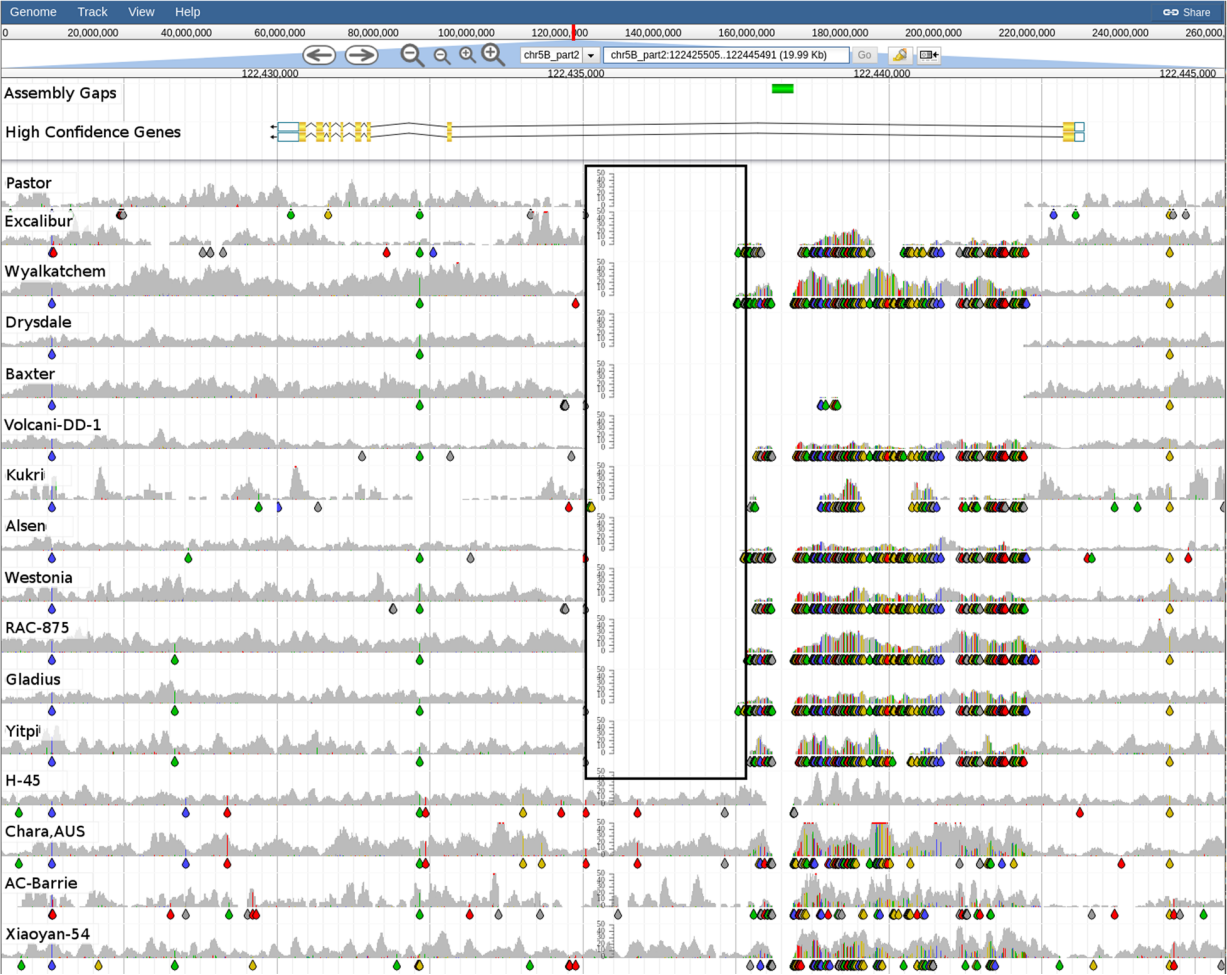
Graphical representation of read coverage and SNPs/indels around *VRN-B1*. The High Confidence gene prediction track (HC Genes) shows the gene models for TraesCS5B01G396600 (*VRN-B1*; chr5B_part2: 122425505-122445491); SNP Coverage tracks for 15 spring types and 1 winter type (Xiaoyan-54). All but 3 spring types (H-45, Chara and AC-Barrie) have a deletion in the first intron of *VRN-B1* as indicated by the lack of read alignment coverage (boxed). Coloured drops hanging off the read coverage profile indicate the presence of putative SNPs/indels to the reference, A-green, T-red, C-blue, G-yellow, indel-grey

**Fig. 5:**
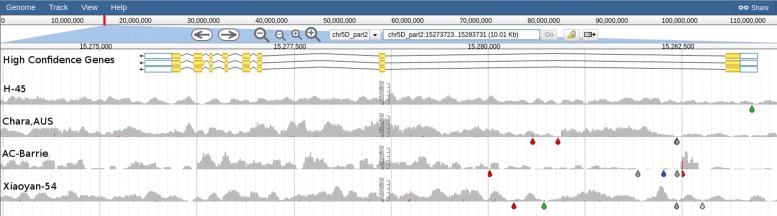
Graphical representation of read coverage and SNPs/indels around *VRN-D1*. The tracks from top to bottom are: High Confidence Gene Predictions showing the gene models for TraesCS5D01G401500 (*VRN-D1*; chr5D_part2:15273724-15283732); SNP Coverage tracks for 1 winter type (Xiaoyan-54) and the 3 spring types (H-45, Chara and AC-Barrie) which do not possess the *VRN-B1* deletion. There is no evidence for a deletion in the three spring types for *VRN-D1*. Coloured drops hanging off the read coverage profile indicate the presence of putative SNPs/indels to the reference, A-green, T-red, C-blue, G-yellow, indel-grey

**Fig. 6:**
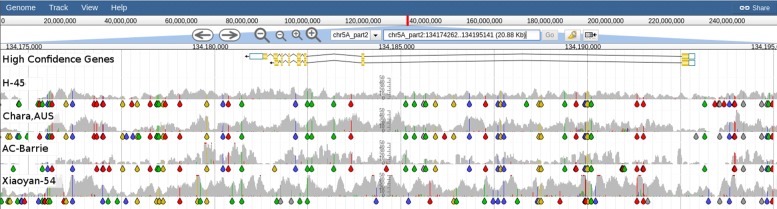
Graphical representation of read coverage and SNPs/indels around *VRN-A1*. The tracks from top to bottom are: High Confidence Gene Predictions showing the gene models for TraesCS5A01G391700 (*VRN-A1*;chr5A_part2:134174264-134195143); SNP Coverage tracks for 1 winter type (Xiaoyan-54) and the 3 spring types (H-45, Chara and AC-Barrie) which do not possess either a *VRN-B1* or *VRN-D1* deletion. Putative SNPs/indels indicate the spring types share the same haplotype which differs from the haplotype of the winter wheat. Coloured drops hanging off the read coverage profile indicate the presence of putative SNPs/indels to the reference, A-green, T-red, C-blue, G-yellow, indel-grey

Using DAWN we were able to see that 12 of the 15 spring wheats in the BPA panel had a *VRN-B1* deletion, as indicated by a lack of read alignment coverage in those accessions (Fig. 4). Three of these 12 spring wheats have evidence for an ≈8 Kbp deletion (Pastor, Drysdale and Baxter) while the other 9 spring wheats seem to have an ≈2.7 Kbp deletion and the remaining ≈5.3 Kbp is significantly different to CS. Of the remaining three spring wheats (H-45, Chara and AC-Barrie) which lack a *VRN-B1* deletion, they also lack a *VRN-D1* deletion (Fig. 5). The variable read alignment coverage around *VRN-D1* and *VRN-A1* make it difficult to determine the precise combination of alleles at these loci (Fig. 6).

#### Wx-B1 nulls

**Fig. 7:**
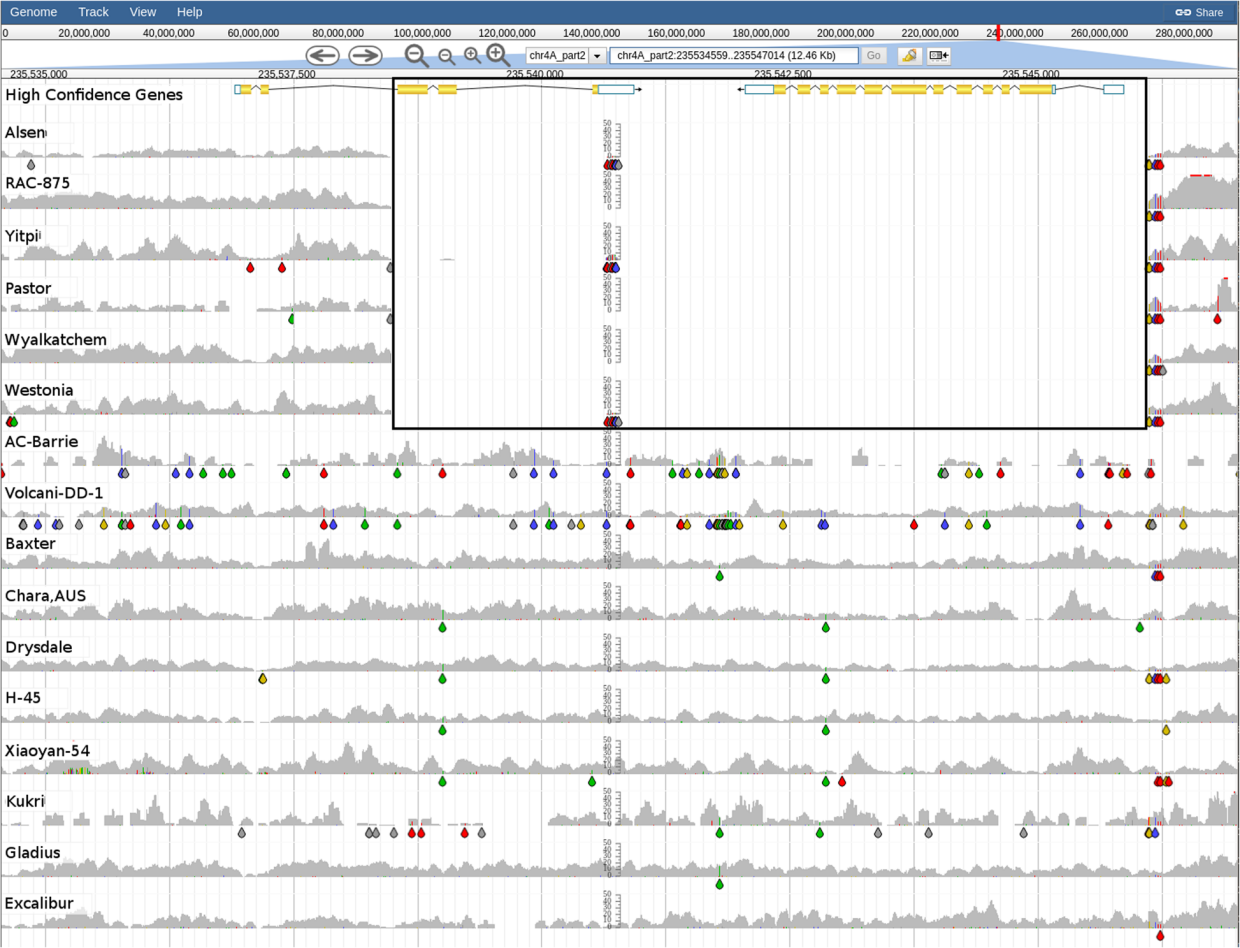
Graphical representation of read coverage and SNPs/indels around the *Wx-B1* locus. The tracks from top to bottom are: High Confidence Gene Predictions showing the gene models for TraesCS4A01G418100 (left) and TraesCS4A01G418200 (*Wx-B1*, right) and SNP Coverage tracks for the 16 BPA accessions (chr4A_part2: 235534559-235547014). Six accessions (Alsen, RAC-875, Yipti, Pastor, Wyalkatchem and Westonia) show evidence of a deletion (boxed) as indicated by the lack of read coverage, while the remaining ten do not have a deletion. Coloured drops hanging off the read coverage profile indicate the presence of putative SNPs/indels to the reference, A-green, T-red, C-blue, G-yellow, indel-grey

The ratio of the two main macromolecules, amylose and amylopectin, is closely related to the quality of starch in the wheat grain, with high amylose being associated with low noodle quality [41]. TraesCS4A01G418200 , also known as “waxy”, encodes a granular bound starch synthase. This gene is solely responsible for the synthesis of amylose in wheat and has three homeologs: *Wx-A1* on chromosome 7A, *Wx-B1* on chromosome 4AL (7B translocation) and *Wx-D1* on chromosome 7D [42]. Null alleles of waxy genes have been described previously in a variety of studies, for instance when examining Mexican [43], Italian [44], Spanish [45] and 324 European accessions which included landraces and spelt wheats [46]. The identification of accessions with these null alleles have allowed the development of new lines with low amylose content. In all cases, *Wx-B1* appeared to be the most polymorphic locus. For a recent review on waxy proteins and their genes see Guzman & Alvarez 2016 [47]. Using DAWN we were able to see a deletion (≈8 Kbp) in six accessions from the BPA panel (Alsen, Pastor, RAC-875, Westonia, Wyalkatchem and Yitpi). This deletion included the whole of *Wx-B1* (Fig. 7) as well as the 3 ^′^ end of a neighbouring gene (TraesCS4A01G418100), annotated as coding for succinate dehydrogenate subunit 5. However, we are not aware of any phenotypic consequences resulting from the partial deletion of this gene. We also observed that *Wx-D1* was conserved across all 16 BPA accessions.

#### TOM1 deletion

Higher plants have two strategies for the uptake of Fe(III) from the rhizosphere (Marschner et al. 1986). The grasses (including wheat, maize, rice and barley) secrete mugineic acid (MA) family phytosiderophores (PS) from their roots into the rhizosphere to chelate and solubilise iron. These iron-PS complexes are taken up into the roots through specific transporters. Transporter of MAs 1 (*TOM1*) has been identified as the likely gene encoding the efflux transporter of 2 ^′^-deoxymugineic acid (DMA) in plants [48]. Rice contains five homologues of *TOM1*, two being in tandem with *TOM1* on chromosome 11 (*TOM2* and *TOM3*) and three others in tandem on chromosome 12 [49]. The function of *TOM3* and the chromosome 12 homologues have not yet been determined, but *TOM2* is thought to be involved in the translocation of metal ions inside the plant body [49].

**Fig. 8:**
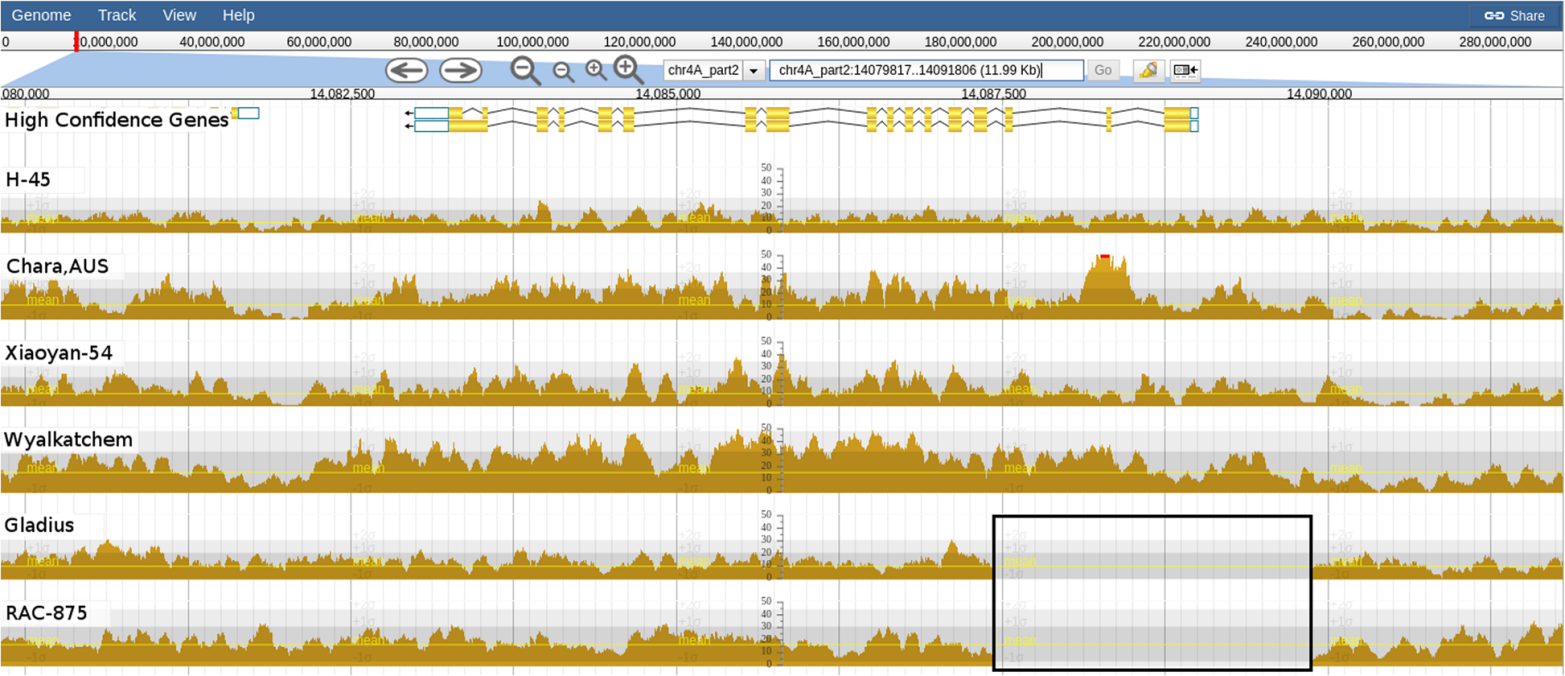
Read alignment coverage across TOM-A1. A 2.5 Kbp deletion is observed in Gladius and RAC-875 (boxed) which covers the first three exons of TOM-A1 (chr4A_part2:14079821-14091810)

We identified the *TOM1* homeologues: *TOM-A1* on chromosome 4A (TraesCS4A01G187500), *TOM-B1* on chromosome 4B (TraesCS4B01G131400) and *TOM-D1* on chromosome 4D (TraesCS4D01G125900). We saw that Gladius and RAC-875 have a deletion (≈2.5 Kbp) which spans the first three exons of *TOM1* and ≈1 Kbp of promoter region (Fig. 8). While this deletion would certainly lead to a *TOM-A1* null, it is not known if these two accessions are more susceptible to iron deficiency or if the *TOM-B1* or *TOM-D1* homeologues compensate for the absence of *TOM-A1*.

### Copy number variation (CNV)

Duplications of genomic loci are known to have played an important role in the evolution of plant genomes and have been linked to disease risk in humans [50]. While it is believed that CNV predominantly affects intergenic regions, there are known CNVs which affect protein-coding genes. For example, CNV has been linked to important traits such as flowering time, plant height and resistance to biotic and abiotic stresses, including boron tolerance in barley [51]. For a recent review of CNV in plants see Zmienko et al. 2014 [52].

**Fig. 9:**
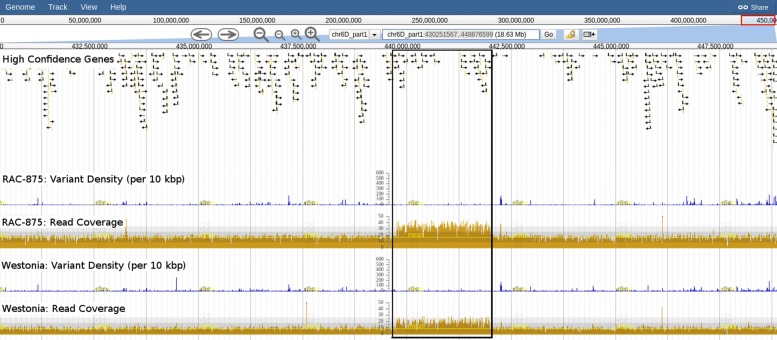
Putative Copy Number Variation (CNV) on chromosome 6D. The putative CNV (boxed) has twice the read coverage depth of the genome in RAC-875 and Westonia. It spans a ≈2.3 Mbp region (chr6D_part1:430251567-448876599) and contains 27 high confidence gene models

Using read coverage depth tracks it is possible to identify putative increases in CNV compared to the CS reference and to delineate the boundaries of the duplication. This is especially the case for the D genome where read coverage depth is less variable. One such example is a ≈2.3 Mbp region on chromosome 6D which shows an ≈2 fold higher coverage (and >2*SD) compared to the mean coverage of the rest of the genome. This putative CNV encompasses 27 high confidence gene models and is only observed for RAC-875 and Westonia (Fig. 9). While Additional file 6 contains coordinates of regions with read coverage >2*SD from the mean, we encourage those with special interest in CNV to analyse our data using the latest computational tools [53].

### Haplotypes

Haplotype blocks are usually defined using linkage disequilibrium (LD) estimated between pairs of markers. Methods to define haplotype blocks require the selection of somewhat arbitrary LD thresholds, especially for species where there is limited information on the extent of LD. DAWN allows haplotype blocks to be visualised using the distribution of SNPs/indels along the chromosomes of the 16 BPA accessions. Different haplotype alleles and recombination between blocks can also be observed.

**Fig. 10:**
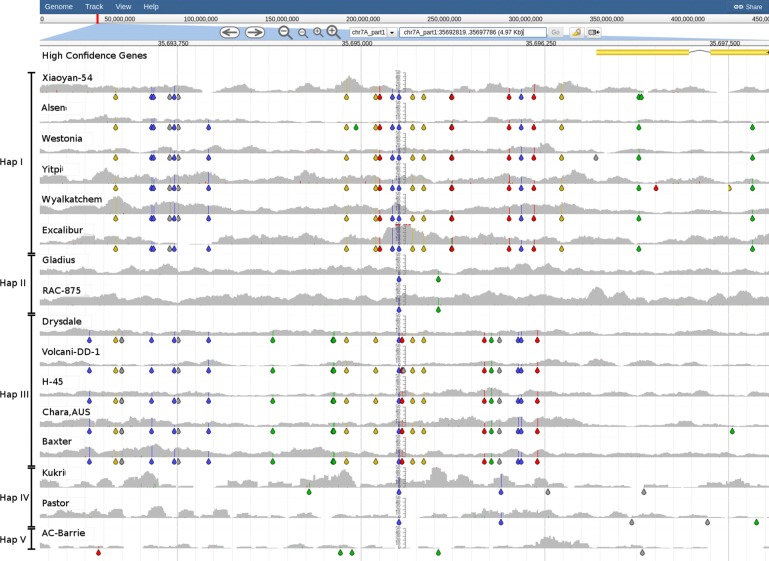
Haplotypes in the BPA accessions on 7A at the phosphate transporter gene TraesCS7A01G070000. The tracks from top to bottom are: Assembly gaps in CS reference; High Confidence Gene Predictions showing the gene model for TraesCS7A01G070000 (chr7A_part1:35692818-35697785). SNP and read coverage tracks for: Xiaoyan-54, Alsen, Westonia, Yipti, Wyalkatchem, Excalibur (first haplotype group), Gladius, RAC-875 (second haplotype), Drysdale, Volcani-DD-1, H-45, Chara, Baxter (third haplotype), Kukri, Pastor (fourth haplotype), AC-Barrie (fifth haplotype). Coloured drops hanging off the read coverage profile indicate the presence of putative SNPs/indels to the reference, A-green, T-red, C-blue, G-yellow, indel-grey

**Fig. 11:**
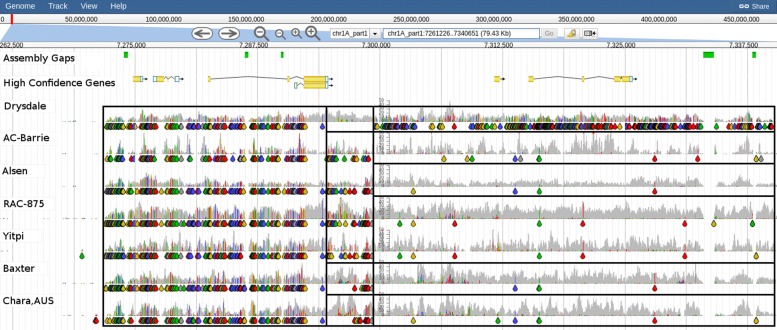
Haplotype changes indicative of recombination break points. SNP Coverage tracks for seven accessions on chr1A_part1:7261243-7340668 . Allowing for poor coverage of some SNPs/indels, accessions have been sorted by haplotypes and haplotype blocks highlighted by boxes. Two putative recombination break points can be inferred by changes in the haplotypes group membership. Coloured drops hanging off the read coverage profile indicate the presence of putative SNPs/indels to the reference, A-green, T-red, C-blue, G-yellow, indel-grey

The nucleotide polymorphisms visible in Fig. 10 allow grouping of the BPA accessions into five distinct haplotypes. Using the information carried in these SNP Coverage tracks, it is possible to see changes in haplotypes as a result of recombination. Fig. 11 shows a region on chromosome 1A in the vicinity of TraesCS1A01G013000 , a gene annotated as being “disease resistance family protein”. The SNP pattern in the region immediately preceding this gene clearly shows that the seven accessions share the same haplotype. However, at about base 7,294,500 (near the 3 ^′^ UTR of this gene) there is a putative recombination break point as evident from the 4 distinct haplotypes immediately following this position: 1) Drysdale; 2) AC-Barrie, Alsen, RAC-875 and Yitpi; 3) Baxter and 4) Chara. A second putative recombination break point at around base 7,300,000 results in additional haplotypes to give 6 distinct haplotypes following this position: 1) Drysdale; 2) AC-Barrie; 3) Alsen; 4) RAC-875 and Yitpi; 5) Baxter and 6) Chara.

As with the example in Fig. 10, we have observed a propensity for putative recombination break points to occur within close proximity of genes. This is consistent with observations made in yeast where the double-strand breaks, which are required for recombination, tend to occur 5 ^′^ of genes near the promoters [54] and in maize where a recombination hotspot was located in the 5 ^′^ transcribed region of the anthocyanin1 (*a1*) gene [55]. Similarly, a recent study of crossover events on wheat chromosome 3B showed a significant association of crossovers with genic features, particularly those which were expressed during meiosis [56].

### Centromeres

It has been known for many years that recombination events are unequally distributed along wheat chromosomes such that their frequencies decrease from telomeres towards the centromeres [57–63]. More recently, Choulet et al. [64] partitioned the pseudomolecule for chromosome 3B with respect to centromere location, gene density and recombination rate and estimated that the centromere extended from 265 Mbp to 387 Mbp.

**Fig. 12:**
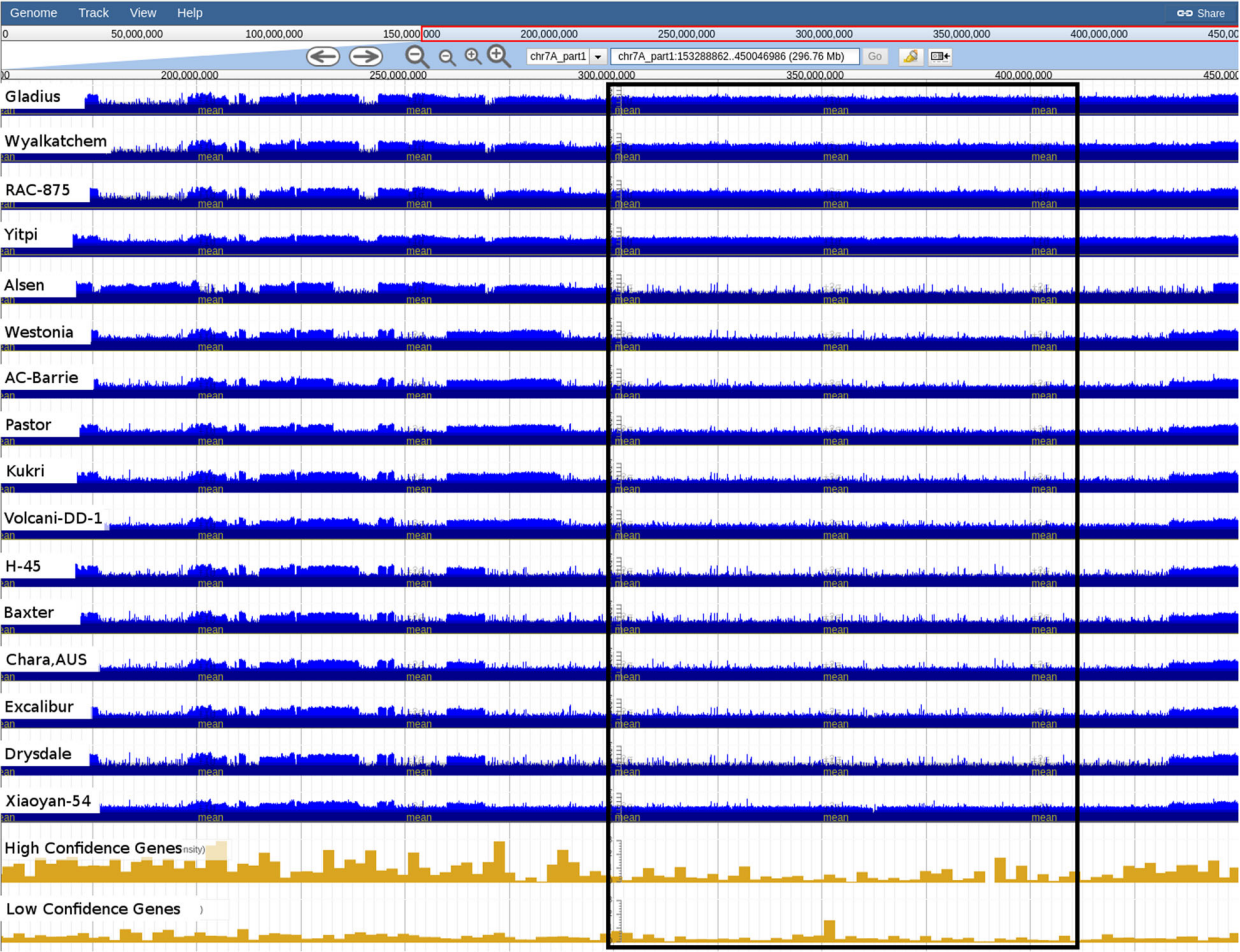
Variant density tracks for chromosome 7A around the predicted centromeric region. Variant density (SNPs/indels per 10 Kbp - log scaled) tracks for 16 accessions, together with high confidence (HC) and low confidence (LC) gene tracks (chr7A_part1:153245006-450046986). A reduction in HC genes coincides with regions of uniform variant density across the accessions. Predicted centromeric region (boxed) is from 300-410 Mbp

To explore whether identification of centromeric regions by visual inspection is possible with DAWN, and investigate the level of diversity across the centromeres among the 16 BPA accessions we first examined the variant call density and concomitant distribution of high and low confidence genes at a megabase scale. Although we expected the centromere to be contained within part1 of each pseudomolecule we analysed the complete length of the pseudomolecules (i.e. part1 and part2). While we observed a reduction of high confidence gene density for most chromosomes (for example Fig. 12), these could be subtle and did not allow us to demarcate the centromeric regions. However, using variant call density tracks we observed a lower number of changes in variant density in part1 compared to part2 of the pseudomolecules. Thus corroborating our expectation that the centromeres are located within part1 of the pseudomolecules.

To determine whether the visual observations indeed coincided with the centromeric regions of the pseudomolecules we analysed previously described centromeric sequences and their distributions along the chromosomes (Additional file 9). Once approximate borders of the putative centromeric regions were established we investigated conservation of haplotypes from the left border to the right border, employing the “Variant Calls” tracks which allows visualization of several 100 Kbp and the “SNP Coverage” tracks for visualizing variants together with read coverage at scales up to 30 Kbp.

Table 2 shows the results of distribution of centromeric sequences and the investigation of the haplotypes across the delimited regions. For a BPA accession to be assigned to a haplotype group it had to be clearly identical with other genotypes. When an accession was similar to another but showed occasional additional variants we did not assign it to a group. In cases where a cultivar was found to be distinct from all other haplotypes, it would form its own single member group, for instance for chromosome 2B, Baxter, AC-Barrie and Xiaoyan-54 all have unique haplotypes. We found varying numbers of discrete haplotypes and groupings for most chromosomes, with the conspicuous exception of the centromeric region of chromosome 3B; all 16 BPA cultivars displayed the same haplotype which was clearly distinct from Chinese Spring. As shown in Table 2 our approximate positioning of the centromere borders occasionally extended beyond observed changes in haplotypes. While a more accurate determination of the centromere regions awaits experimental verification, the fact that the observed haplotype changes are close to our predictions are encouraging.

**Table 2:** Location of centromeres and number of haplotype groups present in BPA accessions

Chr	Start	End	Size	# Haplotype	Haplotype	Groups
	(Mbp)	(Mbp)	(Mbp)	groups	block break	
					points (Mbp)	
1A	160	260	100	2	Xia: 256.967	Group1: Xia, Yit, Exc
					Exc: 258.867	Group2: Kuk, Gla, Wyal, Wes, Bax, H45, Cha, Dry, Pas, RAC, ACB, Vol
1B	180	280	100	5		Group1: Xia
						Group2: Vol
						Group3: ACB, Exc, Als, RAC
						Group4: Kuk, Gla, Wyal, Yit, Wes
						Group5: Bax, Pas, Cha, Dry
1D	146	196	50	2		Group1: Cha, Dry, Exc, Wes, Vol
						Group2: H45, Pas, Wyal, RAC, Xia
2A	270	370	100	3		Group1: ACB, Als, Bax, Dry, Exc, H45, Pas, Kuk, Wyal
						Group2: Cha, RAC, Yit, Gla, Wes, Vol
						Group3: Xia
2B	270	385	115	7	Yit: 379.837	Group1: Bax
						Group2: ACB
						Group3: Xia
						Group4: Wes, Cha, Als
						Group5: RAC, Yit, Gla, Exc
						Group6: Pas, H45, Dry
						Group7: Vol, Wyal, Kuk
2D	220	300	80	3		Group1: Yit, Xia, Als, H45
						Group2: Kuk, Cha, Dry, Pas, RAC, Gla, Exc
						Group3: Bax, Wes, Wyal
3A	240	370	130	4		Group1: Yit, Kuk, Xia, Dry
						Group2: Cha, H45, Bax, Wes, Wyal
						Group3: Gla, RAC
						Group4: Vol, Exc
3B	280	390	110	1		All
3D	200	280	80	2	Bax: 276.588	Group1: Yit, Wes, Bax, Als, Cha, Wyal, Exc
						Group2: Dry, Gla, RAC
4A	245	360	115	3		Group1: Pas, Wes, Yit, H45, Bax
						Group2: Als, Cha, Dry, Wyal, Exc
						Group3: RAC, Gla
4B	270	375	105	6		Group1: ACB
						Group2: RAC, Dry, Kuk, Exc, Wyal
						Group3: Bax, H45, Xia
						Group4: Wes, Gla, Cha
						Group5: Als
						Group6: Yit
4D	194	226	32	4		Group1: RAC, Dry
						Group2: Xia, H45
						Group3: Wyal, Bax
						Group4: Cha, Vol
5A	185	310	125	4		Group1: Als
						Group2: Vol
						Group3: Wyal, Gla, Bax, Dry
						Group4: Yit, Cha, H45, Xia
5B	145	240	95	3		Group1: RAC, Cha, Exc, H45, Dry, Als
						Group2: Wes, Bax, Wyal
						Group3: Yit, Gla
5D	145	245	100	2	RAC: 244.646	Group1: RAC, Gla, H45, Dry, Wes
					Gla: 244.646	Group2: Exc, Wyal
6A	240	355	115	3		Group1: Bax, Cha, H45, Wyal, Wes, Xia
						Group2: Kuk, Als, Dry, Pas, ACB, Yit
						Group3: RAC, Exc, Gla, Vol
6B	270	360	90	2	Exc: 345.417	Group1: Kuk, Xia, Exc, Dry, Wes, Yit
						Group2: Bax, Vol, Als, H45, Pas, Cha, Wyal, RAC, Gla
6D	184	230	46	3		Group1: Vol, Als, Dry, H45
						Group2: Cha, Kuk, Wes, Wyal
						Group3: RAC, Yit, Gla
7A	300	410	110	3		Group1: Vol, ACB, Pas, Wes
						Group2: Exc, Kuk, Bax, Cha, Dry, H45, Xia
						Group3: Wyal, RAC, Yit, Gla
7B	250	315	65	5		Group1: Vol
						Group2: Kuk, Als
						Group3: Yit, Wyal, Exc, Gla, RAC, Wes
						Group4: Dry, H45, Pas, Xia
						Group5: Bax, Cha
7D	300	385	85	4		Group1: Kuk, Xia, Wyal
						Group2: RAC, Exc, Gla
						Group3: Yit, H45, Als, Wes
						Group4: Dry, Bax, Cha (Pas)

The discovery of a single shared haplotype for the centromeric region of 3B present in the BPA accessions is peculiar, since these accessions originate from different regions of the world. Horvath et al. [65] calculated that chromosome 3B had a lower diversity than average for the entire B-genome, but their finding was based on markers located along the whole chromosome, and was not observed in other diversity studies [66, 67]. Cubizolles et al. [68] corroborate our results, they also found only two haplotypes, with the minor haplotype being present in mostly Asian derived lines (possibly the Chinese Spring haplotype). Wheat breeding can impose strong selection pressures in favour of loci encoding disease resistance, contributing to yield or quality [69]. Whether this could be the cause for the low haplotype diversity observed at the 3B centromere warrants further investigation.

### Marker development

QTL positional cloning projects rely on the development of new polymorphic markers that are then used to screen the population under investigation for informative recombinants. Information on SNP/indel positions is the starting point for the design of new high-throughput markers such as KASP^TM^ [70]. DAWN facilitates marker design by providing SNP/indel positions among the 16 BPA accessions as well as allowing the visualisation of previously developed markers such as the 90K SNP array and the 820K Axiom arrays.

The sequences of the markers flanking a QTL can be aligned to the CS IWGSC RefSeq v1.0 using BLASTn to find their position in the reference genome. Alternatively, if the flanking markers are among the data sets already included in DAWN (e.g. Illumina Infinium iSelect 90K and 9K SNP chip, 820K Axiom array), they can be easily located through a search in the DAWN interface. By visualising the QTL interval in DAWN, one can obtain information on the number of predicted genes, the number of haplotypes among the 16 BPA accessions and the SNPs/indels present in the region. For large QTL intervals, markers can be designed and spaced based on the knowledge of the haplotype blocks present in the area, reducing the number of markers to be developed. In the case of small QTL intervals, markers can be designed to target genes in the region or even specific regions of target genes. SNP/indel positions more likely to be polymorphic can also be selected based on the frequency of a particular allele among the 16 BPA accessions.

## Limitations

### Read alignments

Deletion and regions which are highly divergent from Chinease Spring may be almost devoid of read alignments. As such, users are encouraged to investigate further which of these situations is likely to be true. One option is to look for the existance of genes, which fall within such regions, in the wheat pangenome [71, 72].

Short read Illumina data is the predominant sequencing data available today. While Illumina data can be produced at sufficient volumes for sequencing wheat genomes and transcriptomes, it also contains inherent biases and limitations mainly due to the nature of short reads and GC biases [73]. Therefore, regions with high GC bias tend to be under-represented in terms of read coverage depth for both WGS and RNA-Seq data sets, reducing the power to detect variation between accessions.

### Interface

Low and uneven read coverage depth in data sets used for calling variants, leads to missing data in the “Variant Calls” tracks and can mislead the user. However, used together with the “SNP Coverage“ or read coverage depth tracks, one can mitigate this risk by also considering the read coverage depth at a variant site. Care should be taken in overinterpreting the “SNPs” shown in a “SNP Coverage” track as they are based on read alignment missmatches rather than robust variant calling. In addition, they are rendered on-the-fly and so do not scale well, particularly when visualising many accessions over large physical distances. In order to improve the visualisation of coverage and variant information we would look to develop a new JBrowse track capable of rendering read alignment coverage from a BigWig file while superimposing variant information from a VCF file.

## Reference genome to pan-genome

In addition to the IWGSC RefSeq v1.0, two further CS assemblies have been released [21, 74], both of which are a significant improvement over the 2014 published Chromosomal Survey Sequences [75]. We chose IWGSC RefSeq v1.0 as the reference sequence for DAWN because of the availability of pseudomolecules which facilitate identification of diversity at the chromosomal level. We envision that a future consolidation of all CS assemblies will resolve discrepancies and fill gaps, leading to a single CS reference for the community. Until then, the existence of multiple genome assemblies presents a challenge for existing wheat resources [76–79] as it demands a decision on which assembly to use as the reference, or alternatively, to consider all assemblies as a reference. Furthermore, WGS, RNA-Seq and exome capture data will require reprocessing to leverage these improvements and highlights the importance of data sets conforming to FAIR Data Principles [80, 81].

As the number of resequenced genomes increases, the benefits of using a “pan-genome” to represent the genomic repertoire of all the sequenced accessions becomes more apparent [82, 83]. Initial attempts to create a wheat pan-genome have focused on supplementing an existing genome assembly with contigs assembled from reads which had failed to align to the reference genome [71]. Similarly, the rice pan-genome (RPAN) developed for the 3,000 Rice Genome Project (3K RGP) performed a *de novo* assembly of each individual and then removed redundancy to derive a set of sequences that constitute the rice pan-genome [84]. While these approaches provide a convenient linear representation of a pan-genome, it cannot easily be extended to iteratively include newly sequenced genomes without significant effort. While graph representations of the pan-genome are an attractive way to represent the genetic variation which exists between individuals, their utility is hampered by the lack of tools which can utilise these data structures for read alignments, variant calling and visualisation [85]. We expect the future will see a paradigm shift away from linear representations of a (pan-)genome to more sustainable graph based representations [86].

## Conclusions

Through DAWN we have removed the burden of analysing the whole genome sequencing data of 16 accessions and made these valuable data sets easily accessible to the community through a JBrowse interface in the context of the newly released IWGSC RefSeq v1.0 genome assembly and annotations. By providing examples, we have shown how DAWN can be utilised by researchers to: a) discover diversity of different types among genomes; b) find explanations for reduced recombination; c) identify markers for tracking important traits and d) identify candidate genes under QTL. The example where we explored possible explanations for the tight linkage between *Rlnn1* and yellow flour colour shows the power of visualisation of genomic data at different resolutions; it took little time to place the wri-markers onto chromosome 7A and find a possible explanation for experimental observations. Moreover, DAWN could also be used in a predictive way, accelerating research direction and discovery. As more wheat genomes are resequenced and other genomic resources are made available, we can make these available through DAWN. Our processed data sets, which include BAM and VCF files, are made freely available for others to use and explore.

## Availability and requirements

We provide convenient access to the computed data files via a JBrowse interface available via http://crobiad.agwine.adelaide.edu.au/dawn/.

- **Project name:D**iversity **A**mong **W**heat ge**N**omes (DAWN)
- **Project home page:** http://crobiad.agwine.adelaide.edu.au/dawn/
- **Operating system(s):** Platform independent
- **Other requirements:** HTML5 compatible web browser
- **License:** GNU GPL

## Additional files

Additional file 1 IWGSC RefSeq v1.0 annotation preprocessing shell script. Shell script to download, validate and modify IWGSC RefSeq v1.0 annotation files, including splitting GFF3 files based on boundaries defined by the IWGSC. (SH 9 kb)

Additional file 2 Perl helper script for splitting GFF3 files. Perl script for splitting a GFF3 file at chromosomal coordinates defined in a BED file. This converts GFF3 coordinates from full length pseudomolecules to their “parts” and is called by the preprocessing shell script (Additional file 1). (PL 3 kb)

Additional file 3 Script for merging functional annotations into gene models. Shell script to merge functional annotation information into the High and Low confidence gene model GFF3 files. (SH 2 kb)

Additional file 4 Awk helper script for creating correctly formatted GFF3 attribute information. Awk script for generating a file of correctly formatted GFF3 attributes of functional annotation information. It is called by the shell script for merging functional annotations (Additional file 3). (AWK 1 kb)

Additional file 5 MAPQ distribution table. Table showing MAPQ distribution for each WGS accession. Number of aligned reads with a given MAPQ are show together with the cumulative sum of reads ≥ to a given MAPQ (expressed both as a percentage of the aligned read as well as the raw reads). (TAB 28 kb)

Additional file 6 ZIP file containing BED5 format files of high and low read coverage regions. BED5 format file for each WGS accession. The score column (5th column) indicates if the feature (row) represents a region which has >2*SD above (High) or below (Low) the mean read coverage. (ZIP 1455 kb)

Additional file 7 ZIP file containing BED5 format files of high and low variant density regions. BED5 format file for each WGS accession. The score column (5th column) indicates if the feature (row) represents a region which has >2*SD above (High) or below (Low) the mean variant density. (ZIP 526 kb)

Additional file 8 Script to generate data files required for the DAWN data tracks. A script containing all the commands needed to generate all the data files used in DAWN. (SH 9667 kb)

Additional file 9 Materials and Methods. A description of materials and methods used. (PDF 229 kb)

## Abbreviations

BAM: A binary representation of the sequence alignment map (SAM) file format
CS: Chinese Spring
DAWN: Diversity among wheat geNomes
EST: Expressed sequence tag
IWGSC: International wheat genome sequencing consortium
SNP: Single nucleotide polymorphism
SSR: Simple sequence repeat (a.k.a. microsatellite, STR: short tandem repeats)
VCF: Variant call format
WGS: Whole genome shotgun
