# Supplementary material for: DAWN: a resource for yielding insights into the diversity among wheat genomes
Source: BMC Genomics. 2018 Dec 17;19:941. doi: 10.1186/s12864-018-5228-2 (PMC6296097; doi:10.1186/s12864-018-5228-2)
Supplement: Supplementary file 9 — Materials and Methods. A description of materials and methods used. (PDF 229 kb) [file 12864_2018_5228_MOESM9_ESM.pdf]

DAWN: A Resource for Yielding Insights into the Diversity Among Wheat Genomes

Nathan S. Watson-Haigh, Radoslaw Suchecki, Elena Kalashyan, Melissa Garcia & Ute Baumann

Jun 2018

This file contains a more detailed description of various M&M used and some example code for key steps in the analaysis of the data. While these commands are not the *actual* commands executed, they are illustrative of the parameters used. For the full set of commands executed, please see the `commands.sh` file.

WGS Resequencing Data

The quality trimming of the raw paired-end BPA data was performed by removing reads which: contained more than 10% Ns, had greater than 75% homonucleotide runs (A or T, G, C), an average read quality of less than 23, contained adapter sequences (24bp or longer), had sequence-identical mates, were shorter than 50bp after trimming off low quality bases (Q<23) from both ends.

The QC'd data comprises 2x100 bp paired-end Illumina reads totalling approx. 1,425 Gbp (2.3 Tbytes of compressed FASTQ files) of quality filtered reads; a data set size equivilent to almost 1/3 of the 1000 Genomes Pilot Project for Humans (Clarke et al. 2012). Briefly, the data was aligned to the reference genome using minimap2 and reads aligned as proper pairs (max fragment length 1000 bp) with a mapping quality score >=5 were retained. BAM files corresponding to the same accession were subsequently merged to yield a single BAM file per accession.

Read coverage bigWig files were created from reads with mapping quality >=20. These were then processed to identify genomic regions with coverage more than 2*SD from the mean (counting zero coverage as being more than 2*SD less than the mean). Regions more than 2*SD above the mean are candidate duplications vs CS, while those more than 2*SD below the mean (plus zero coverage) are candidate deletions vs CS. Candidate duplication regions <=500 bp apart were merged together and filtered to retain those regions >= 5 kbp. Similarly, candidate deletion regions <=500 bp apart were merged together and filtered to retain those regions >= 50 kbp.

Example Commands Used

```
# Index reference genome for Minimap2
minimap2 -I 50G \
  -d references/161010_Chinese_Spring_v1.0_pseudomolecules_parts.fasta.gz.mmi \
  references/161010_Chinese_Spring_v1.0_pseudomolecules_parts.fasta.gz \
  2>&1 > references/161010_Chinese_Spring_v1.0_pseudomolecules_parts.fasta.gz.mmi.log

# Example: Minimap2 alignment (per pair of read files)
minimap2 -a -x sr -t 1 -2 -K 1G -F 1000 \
  -R "@RG\tID:Alsen/Alsen_C4MH4ACXX_NoIndex_L002\tLB:Unknown\tPL=Illumina\tPU=Alsen_C4MH4ACXX_NoIndex_L002\tSM:Alsen" \
  references/161010_Chinese_Spring_v1.0_pseudomolecules_parts.fasta.gz.mmi \
  Alsen/Alsen_C4MH4ACXX_NoIndex_L002_R1.qc.fastq.gz \
  Alsen/Alsen_C4MH4ACXX_NoIndex_L002_R2.qc.fastq.gz \
  | samtools view -ub -f 2 -q 5 -- \
  | samtools sort -T "/tmp" --threads 10 -m 5G \
  | samtools calmd -b --threads 1 - "references/161010_Chinese_Spring_v1.0_pseudomolecules_parts.fasta.gz" \
  > mapped_reads/Alsen/Alsen_C4MH4ACXX_NoIndex_L002.bam

# Example: Read coverage bigWig creation (per chr part per accession)
samtools depth \
  -Q 20 \
  --reference references/161010_Chinese_Spring_v1.0_pseudomolecules_parts.fasta \
  by_chr/chr1D_part1/Alsen.realigned.bam \
  | mawk -f scripts/depth2bedgraph.awk \
  > "/tmp/file.bam.bedgraph"
bedGraphToBigWig \
  "/tmp/file.bam.bedgraph" \
  references/161010_Chinese_Spring_v1.0_pseudomolecules_parts.fasta.chrom.sizes \
  "by_chr/chr1D_part1/Alsen.realigned.bam.bw"
```

Variant Calling and Variant Density

Variant calling was performed following indel realignment (using GATK) using SAMtools and BCFtools (Li 2011). This process was parallelised in two ways: 1) by chromosome parts and then using Sambamba for parallelising variant calling on each chromosome part.

Only reads with a mapping quality of >=20 and in the `rf` orientation were included in the pileup stage and thus contribute to variant calling by BCFtools using "call –consensus-caller –variants-only". Soft filtering was then performed by BCFtools to annotate the FILTER field in the VCF files as follows:

	Homozygous (GT="hom")	Heterozygous (GT="het")
Low Quality (QUAL<30)	LowQualHom	LowQualHet
High Quality (QUAL>=30)	PASS	Het

Example Commands Used

```
# Example: Indentify indel regions for realignment (per chr partper accession)
java -Xms1G -Xmx18G -jar "${EBROOTGATK}/GenomeAnalysisTK.jar" \
  --analysis_type RealignerTargetCreator \
  --reference_sequence references/161010_Chinese_Spring_v1.0_pseudomolecules_parts.fasta \
  --input_file mapped_reads_merged/Alsen.bam \
  --intervals chr1D_part1 \
  --num_threads 1 \
  --maxRuntime 2 --maxRuntimeUnits HOURS \
  --performanceLog logs/find_indel_regions/chr1D_part1/Alsen.bam.gatk_intervals.performance \
  --out by_chr/chr1D_part1/Alsen.bam.gatk_intervals.list

# Example: Realign identified indel regions (per chr partper accession)
java -Xms1G -Xmx1G -Djava.io.tmpdir=/tmp -jar "${EBROOTGATK}/GenomeAnalysisTK.jar" \
  --analysis_type IndelRealigner \
  --input_file mapped_reads_merged/Alsen.bam \
  --intervals chr1D_part1 \
  --reference_sequence references/161010_Chinese_Spring_v1.0_pseudomolecules_parts.fasta \
  --targetIntervals by_chr/chr1D_part1/Alsen.bam.gatk_intervals.list \
  --consensusDeterminationModel USE_READS \
  --LODThresholdForCleaning 0.4 \
```

```
  --out by_chr/chr1D_part1/Alsen.realigned.bam \
  --num_threads 1 --num_cpu_threads_per_data_thread 1 \
  --performanceLog logs/realign_indels/chr1D_part1/Alsen.realigned.bam.gatk_realign.performance

# Example: Variant calling (per chr partper accession)
sambamba mpileup --nthreads 1 \
  by_chr/chr1D_part1/Alsen.realigned.bam \
  --samtools " --fasta-ref references/161010_Chinese_Spring_v1.0_pseudomolecules_parts.fasta --uncompressed --min-MQ 20 --rf 2" \
  --bcftools "call --consensus-caller --variants-only" \
  | bcftools filter --soft-filter LowQualHom --exclude '%QUAL<30 && GT="hom"' \
  | bcftools filter --soft-filter LowQualHet --exclude '%QUAL<30 && GT="het"' \
  | bcftools filter --soft-filter Het --exclude '%QUAL>=30 && GT="het"' \
  | bgzip \
  > by_chr/chr1D_part1/Alsen.realigned.bam.vcf.gz

# Example: Variant density bigWig over 10 kbp disjoint windows (per chr partper accession)
bedtools makewindows \
  -g references/161010_Chinese_Spring_v1.0_pseudomolecules_parts.fasta.chrom.sizes \
  -w 10000 -s 10000 \
  > references/161010_Chinese_Spring_v1.0_pseudomolecules_parts.fasta.windows_w10000_s10000.bed
bedtools coverage \
  -a references/161010_Chinese_Spring_v1.0_pseudomolecules_parts.fasta.windows_w10000_s10000.bed \
  -b by_chr/chr1D_part1/Alsen.realigned.bam.vcf.gz \
  -counts \
  > "/tmp/file.bam.bedgraph"
bedGraphToBigWig "/tmp/file.bam.bedgraph" \
  references/161010_Chinese_Spring_v1.0_pseudomolecules_parts.fasta.chrom.sizes \
  by_chr/chr1D_part1/Alsen.realigned.bam.vcf.w10000_s10000.bw
```

Gene Expression Data

The data consisted of 1.4 billion pairs of 100 bp paired-end Illumina reads, totalling approx. 277 Gbp (232 GBytes of compressed FASTQ files).

Chinese Spring reads were aligned to the reference genome using STAR. Alignment parameters were set to force reads to align perfectly across their full

length and to have <5 putative alignment positions. BAM files from replicate samples were merged to yield a single BAM files per tissue and stage (15 in total).

Example Commands Used

```
# Index reference genome for STAR
awk '{tot+=$2}END{print tot}' references/161010_Chinese_Spring_v1.0_pseudomolecules_parts.fasta.fai \
  > references/161010_Chinese_Spring_v1.0_pseudomolecules_parts.fasta.gz_STAR_index/GenomeLength
wc -l \
  < references/161010_Chinese_Spring_v1.0_pseudomolecules_parts.fasta.fai \
  > references/161010_Chinese_Spring_v1.0_pseudomolecules_parts.fasta.gz_STAR_index/NumberOfReferences

GENOME_LENGTH=$(cat references/161010_Chinese_Spring_v1.0_pseudomolecules_parts.fasta.gz_STAR_index/GenomeLength)
N_REF=$(cat references/161010_Chinese_Spring_v1.0_pseudomolecules_parts.fasta.gz_STAR_index/NumberOfReferences)
genomeChrBinNbits=$(echo "v=l(${GENOME_LENGTH}/${N_REF}) / l(2); scale=0; def=18; if (def<v) def else v/1" | bc -l)
STAR \
  --runMode genomeGenerate \
  --outTmpDir /tmp/ \
  --genomeDir references/161010_Chinese_Spring_v1.0_pseudomolecules_parts.fasta.gz_STAR_index/ \
  --genomeChrBinNbits ${genomeChrBinNbits} \
  --genomeFastaFiles references/161010_Chinese_Spring_v1.0_pseudomolecules_parts.fasta \
  --limitGenomeGenerateRAM 320000000000 \
  --runThreadN 1

# Example: STAR alignment (per pair of read files)
STAR \
  --runMode alignReads \
  --outStd BAM_SortedByCoordinate \
  --outBAMcompression 0 \
  --outTmpDir /tmp/tmp.XXXXXXXXXX_STARtmp/ \
  --genomeDir references/161010_Chinese_Spring_v1.0_pseudomolecules_parts.fasta.gz_STAR_index \
  --genomeLoad NoSharedMemory \
  --runThreadN 1 \
  --outBAMsortingThreadN 14 \
  --limitIObufferSize 150000000 \
```

```
  --limitBAMsortRAM 190000000000 \
  --readFilesIn spike_Z32/spike_Z32_rep2.1.paired.fastq.gz spike_Z32/spike_Z32_rep2.2.paired.fastq.gz \
  --readFilesCommand pigz -dcp2 \
  --outFileNamePrefix mapped_reads/spike_Z32/spike_Z32_rep2. \
  --outSAMtype BAM SortedByCoordinate \
  --outFilterMultimapScoreRange 0 \
  --outFilterMultimapNmax 5 \
  --outFilterMismatchNoverLmax 0.02 \
  --outFilterMatchNminOverLread 0.98 \
  --outSJfilterOverhangMin 35 20 20 20 \
  --outSJfilterCountTotalMin 10 3 3 3 \
  --outSJfilterCountUniqueMin 5 1 1 1 \
  --alignEndsType Local \
  --alignSoftClipAtReferenceEnds No \
  --outSAMstrandField intronMotif \
  --outSAMattributes All \
  --alignIntronMax 10000 \
  --alignMatesGapMax 10000 \
  --outSAMattrRGline ID:mapped_reads/spike_Z32/spike_Z32_rep2.SJ.out.tab PL:Illumina PU:Unknown LB:Unknown SM:spike_Z32 \
  | samtools calmd -b --threads 1 - references/161010_Chinese_Spring_v1.0_pseudomolecules_parts.fasta.gz \
  > mapped_reads/spike_Z32/spike_Z32_rep2.Aligned.sortedByCoord.out.bam

# Example: bigWig creation (per chr part per accession)
samtools depth \
  -Q 20 \
  --reference references/161010_Chinese_Spring_v1.0_pseudomolecules_parts.fasta \
  by_chr/chr1D_part1/spike_Z32.bam \
  | mawk -f scripts/depth2bedgraph.awk \
  > "/tmp/file.bam.bedgraph"
bedGraphToBigWig \
  "/tmp/file.bam.bedgraph" \
  references/161010_Chinese_Spring_v1.0_pseudomolecules_parts.fasta.chrom.sizes \
  "by_chr/chr1D_part1/spike_Z32.bam.bw"
```

Centromeres

Putative centromeric regions for each of the 21 chromosomes was determined by blastN of the following query sequences against IWGSC RefSeq v1.0: (a) centromeric retrotransposon repeats (Genbank: AB048243, AB048244, AB048245) discovered by (Fukui et al. 2001) (b) centromeric FISH clones (pT103 to pTA-k609) identified by (Komuro et al. 2013) and (c) the Quinta elements described by (Choulet et al. 2014). Putatitive positions of the centromeres were deduced from the distribution frequency of the Blast hits when plotted along the pseudomolecules.

Software Environment Used

The following software and versions were used:

- HTSlib v1.8 ("Homepage: HTSlib," n.d.)
- SAMtools v1.8 (Li et al. 2009)
- BCFtools v1.8 ("Homepage: BCFtools V1.8," n.d.)
- minimap2 v2.10 (Li 2018)
- STAR v2.6.0c (Dobin et al. 2013)
- GATK v3.8-1-0 (McKenna et al. 2010)
- picard v2.9.2 ("Homepage: Picard," n.d.)
- BEDTools v2.27.0 (Quinlan and Hall 2010)
- kentUtils v365 (Kent et al. 2002)
- FASTX-Toolkit v0.0.14 ("Homepage: FASTX-Toolkit," n.d.)
- Sambamba v0.6.7 (v0.6.5 used for variant calling commands) (Tarasov et al. 2015)
- Java v1.8.0_74
- pigz v2.4 ("Homepage: Pigz," n.d.)
- parallel v20170922 (Tange 2011)
- mawk v1.2 ("Homepage: Mawk," n.d.)

References

Choulet, Frédéric, Adriana Alberti, Sébastien Theil, Natasha Glover, Valérie Barbe, Josquin Daron, Lise Pingault, et al. 2014. "Structural and Functional Partitioning of Bread Wheat Chromosome 3B." *Science* 345 (6194). American Association for the Advancement of Science. doi:10.1126/science.1249721.

Clarke, Laura, Xiangqun Zheng-Bradley, Richard Smith, Eugene Kulesha, Chunlin Xiao, Iliana Toneva, Brendan Vaughan, et al. 2012. "The 1000 Genomes

Project: Data Management and Community Access.” *Nature Methods* 9 (Apr). The Author(s) SN -: 459 EP. http://dx.doi.org/10.1038/nmeth.1974.

Dobin, Alexander, Carrie A. Davis, Felix Schlesinger, Jorg Drenkow, Chris Zaleski, Sonali Jha, Philippe Batut, Mark Chaisson, and Thomas R. Gingeras. 2013. “STAR: Ultrafast Universal RNA-Seq Aligner.” *Bioinformatics* 29 (1): 15–21. doi:10.1093/bioinformatics/bts635.

Fukui, Kazu-Nori, Go Suzuki, Evans S. Lagudah, Sadequr Rahman, Rudi Appels, Maki Yamamoto, and Yasuhiko Mukai. 2001. “Physical Arrangement of Retrotransposon-Related Repeats in Centromeric Regions of Wheat.” *Plant and Cell Physiology* 42 (2): 189–96. doi:10.1093/pcp/pce026.

“Homepage: BCFtools V1.8.” n.d. http://www.htslib.org/doc/bcftools-1.8.html.

“Homepage: FASTX-Toolkit.” n.d. http://hannonlab.cshl.edu/fastx_toolkit/.

“Homepage: HTSlib.” n.d. http://www.htslib.org/.

“Homepage: Mawk.” n.d. http://invisible-island.net/mawk/.

“Homepage: Picard.” n.d. http://broadinstitute.github.io/picard/.

“Homepage: Pigz.” n.d. https://zlib.net/pigz/.

Kent, W. James, Charles W. Sugnet, Terrence S. Furey, Krishna M. Roskin, Tom H. Pringle, Alan M. Zahler, Haussler, and David. 2002. “The Human Genome Browser at UCSC.” *Genome Research* 12 (6): 996–1006. doi:10.1101/gr.229102.

Komuro, Shirabe, Ryota Endo, Kaori Shikata, and Akio Kato. 2013. “Genomic and Chromosomal Distribution Patterns of Various Repeated DNA Sequences in Wheat Revealed by a Fluorescence in Situ Hybridization Procedure.” *Genome* 56 (3): 131–37. doi:10.1139/gen-2013-0003.

Li, Heng. 2011. “A Statistical Framework for SNP Calling, Mutation Discovery, Association Mapping and Population Genetical Parameter Estimation from Sequencing Data.” *Bioinformatics* 27 (21): 2987–93. doi:10.1093/bioinformatics/btr509.

———. 2018. “Minimap2: Pairwise Alignment for Nucleotide Sequences.” *Bioinformatics*, bty191. doi:10.1093/bioinformatics/bty191.

Li, Heng, Bob Handsaker, Alec Wysoker, Tim Fennell, Jue Ruan, Nils Homer, Gabor Marth, Goncalo Abecasis, Richard Durbin, and 1000 Genome Project Data Processing Subgroup. 2009. “The Sequence Alignment/Map Format and SAMtools.” *Bioinformatics* 25 (16): 2078–9. doi:10.1093/bioinformatics/btp352.

McKenna, Aaron, Matthew Hanna, Eric Banks, Andrey Sivachenko, Kristian Cibulskis, Andrew Kernytsky, Kiran Garimella, et al. 2010. “The Genome Analysis Toolkit: A MapReduce Framework for Analyzing Next-Generation DNA Sequencing Data.” *Genome Research* 20 (9): 1297–1303. doi:10.1101/gr.107524.110.

Quinlan, Aaron R., and Ira M. Hall. 2010. “BEDTools: A Flexible Suite of Utilities for Comparing Genomic Features.” *Bioinformatics* 26 (6): 841–42. doi:10.1093/bioinformatics/btq033.

Tange, O. 2011. “GNU Parallel - the Command-Line Power Tool.”*;Login: The USENIX Magazine* 36 (1). Frederiksberg, Denmark: 42–47. http:

//www.gnu.org/s/parallel.

Tarasov, Artem, Albert J. Vilella, Edwin Cuppen, Isaac J. Nijman, and Pjotr Prins. 2015. “Sambamba: Fast Processing of NGS Alignment Formats.” *Bioinformatics* 31 (12): 2032–4. doi:10.1093/bioinformatics/btv098.
